# Minimally invasive repair of incarcerated foramen of Winslow hernia: first report using a robotic-assisted approach

**DOI:** 10.1093/jscr/rjaf738

**Published:** 2025-09-13

**Authors:** Abanoub Awad, Mitchell Meagher, Joseph Wildenberg, Merna Zaki, Jason Beckermann

**Affiliations:** Department of Surgery, Mayo Clinic Health System, 1400 Bellinger, Eau Claire, WI 54703, United States; Department of Surgery, Mayo Clinic Health System, 1400 Bellinger, Eau Claire, WI 54703, United States; Department of Radiology, Mayo Clinic Health System, 1400 Bellinger St, Eau Claire, WI 54703, United States; Patient Care Specialist, Mayo Clinic Health System, 733 W. Clairemont Ave., Eau Claire, WI 54701, United States; Department of Surgery, Mayo Clinic Health System, 1400 Bellinger, Eau Claire, WI 54703, United States

**Keywords:** foramen of Winslow hernia, internal hernia, robotic-assisted surgery, minimally invasive surgery, robot, case report

## Abstract

Foramen of Winslow hernia is a rare subtype of internal hernias, accounting for ⁓8% of all internal hernia cases. Its nonspecific clinical presentation often delays diagnosis, with ˂10% identified preoperatively. With advances in minimally invasive techniques, robotic-assisted surgery has emerged as a safe and precise method for managing complex intra-abdominal pathology. A 70-year-old male presented with an acute abdomen. He was hemodynamically stable, and computed tomography imaging suggested an internal hernia. Robotic-assisted abdominal exploration revealed a segment of colon herniated through the foramen of Winslow. The colon was carefully reduced without complications. A piece of omentum was placed into the defect, and the retracted gall bladder naturally positioned itself within the foramen, potentially minimizing the risk of recurrence. The patient recovered uneventfully and discharged on postoperative day one. To our knowledge, this is the first documented case of foramen of Winslow hernia repair using a robotic-assisted approach.

## Introduction

Internal hernias are an uncommon cause of intestinal obstruction, accounting for only 0.2%–0.9% of all cases, and are often difficult to diagnose preoperatively due to their nonspecific presentation [1]. Foramen of Winslow hernia is a rare subtype, accounting for ⁓8% of internal hernia cases [2].

The foramen of Winslow, also known as the epiploic or omental foramen, is a normal orifice that communicates the greater and lesser peritoneal sacs [3]. Anatomically, the foramen of Winslow is bordered anteriorly by the hepatoduodenal ligament, posteriorly by the inferior vena cava, superiorly by the caudate lobe of the liver, and inferiorly by the first portion of the duodenum [4]. Normally, the foramen remains sealed due to intra-abdominal pressure, making spontaneous herniation uncommon. Certain anatomical variations, such as an enlarged foramen, long bowel mesentery, redundant colon, or a Riedel’s lobe of the liver, may predispose patients to this type of hernia [5]. Two-thirds of reported cases involved herniation of the ileum followed by a mobile cecum or ascending colon [6].

Diagnosis is often delayed, with ˂10% of cases identified preoperatively [2]. Similar to other internal hernias, delayed diagnosis of foramen of Winslow hernia can result in strangulated or closed-loop obstructions, which, if left untreated, contribute to a high mortality rate approaching 50% [7]. The introduction of robotic surgical systems has offered multiple technical advantages over traditional laparoscopy, including superior magnification, improved visualization, greater instrument handling, and enhanced precision in tissue handling [8].

## Case report

A 70-year-old male patient with hypertension and no prior abdominal surgeries presented to the emergency department with diffuse abdominal pain, mainly in the epigastric region. The pain had started ⁓8 h prior to presentation and was not associated with vomiting or constipation. On physical examination, the patient was hemodynamically stable, and the abdomen was soft and non-distended.

**Figure 1:**
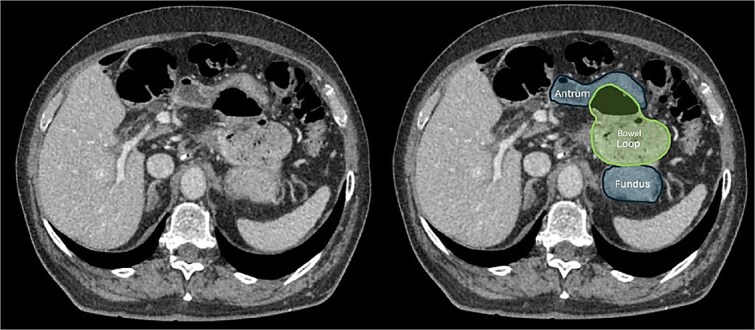
CT scan of the abdomen showing that the colon passing above and to the left of the lesser curvature of the stomach, implying that it had herniated through the foramen of Winslow and into the lesser sac.

A contrast-enhanced computed tomography (CT) scan of the abdomen and pelvis initially suggested small bowel telescoping in the left upper quadrant above the duodenum, with superior displacement of the stomach (Fig. 1). These findings were most consistent with an internal hernia. Additionally, fecalized contents within the herniated bowel suggested delayed transit, likely indicating a partial obstruction.

Given the radiologic findings, the decision was made to proceed with robotic-assisted abdominal exploration to reduce the hernia and assess bowel viability. The procedure was performed using the Da Vinci Xi robotic system and lasted ⁓62 min.

**Figure 2:**
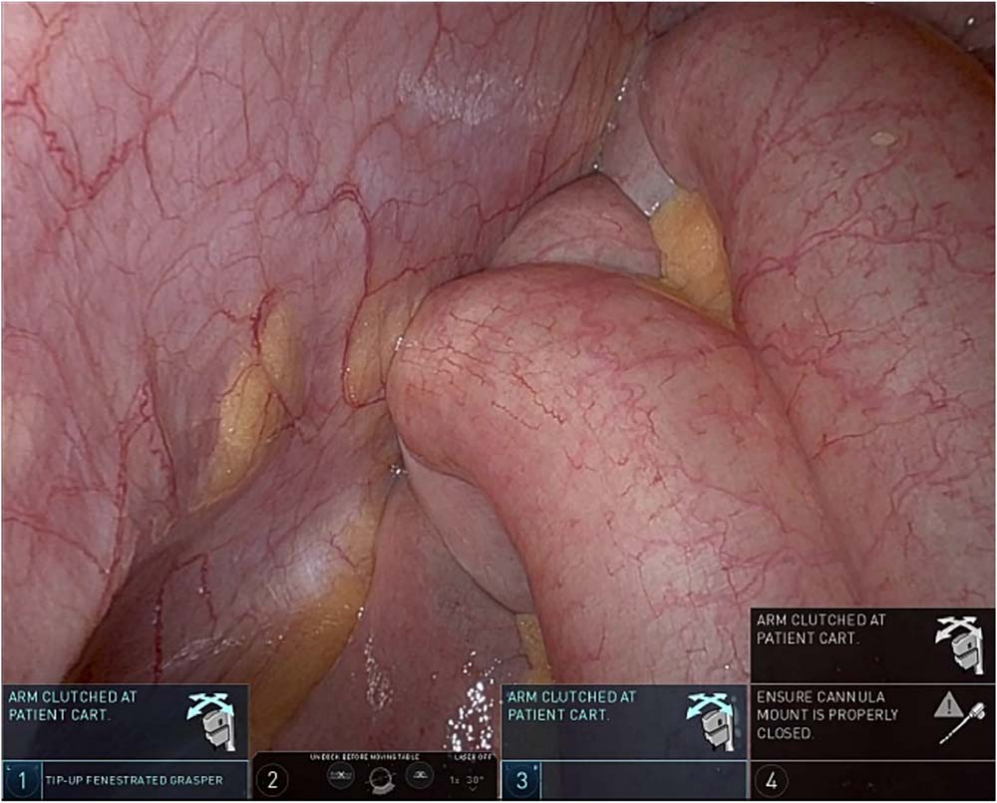
Dilated small bowel loops.

**Figure 3:**
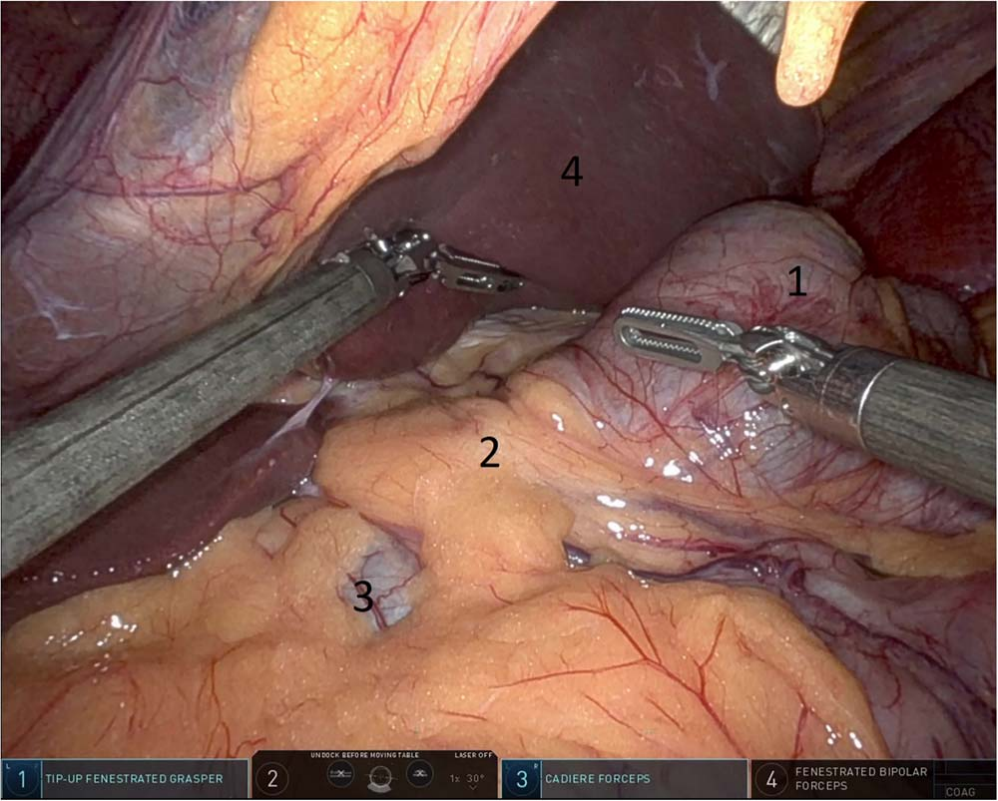
Herniated colon through the foramen of Winslow (1: incarcerated colon, 2: hepato-duodenal ligament, 3: herniating colon, 4: the liver).

**Figure 4:**
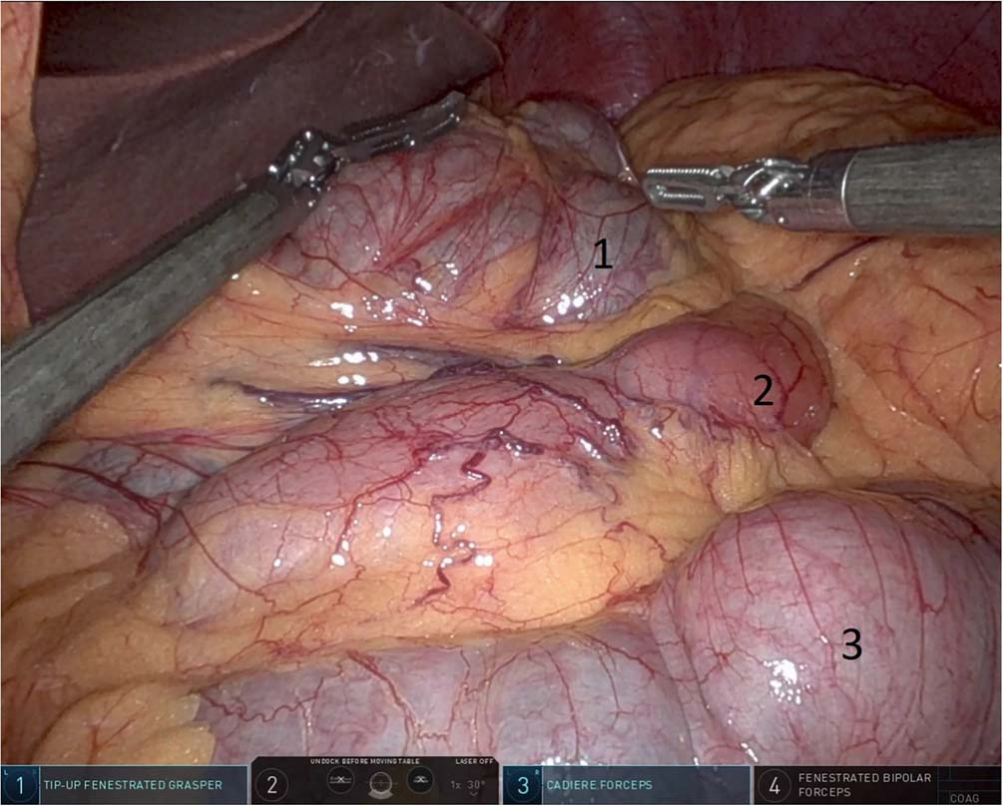
Pars flaccida covering the herniated bowel (1: pars flaccida over the herniated bowel, 2: the stomach, 3: transverse colon).

**Figure 5:**
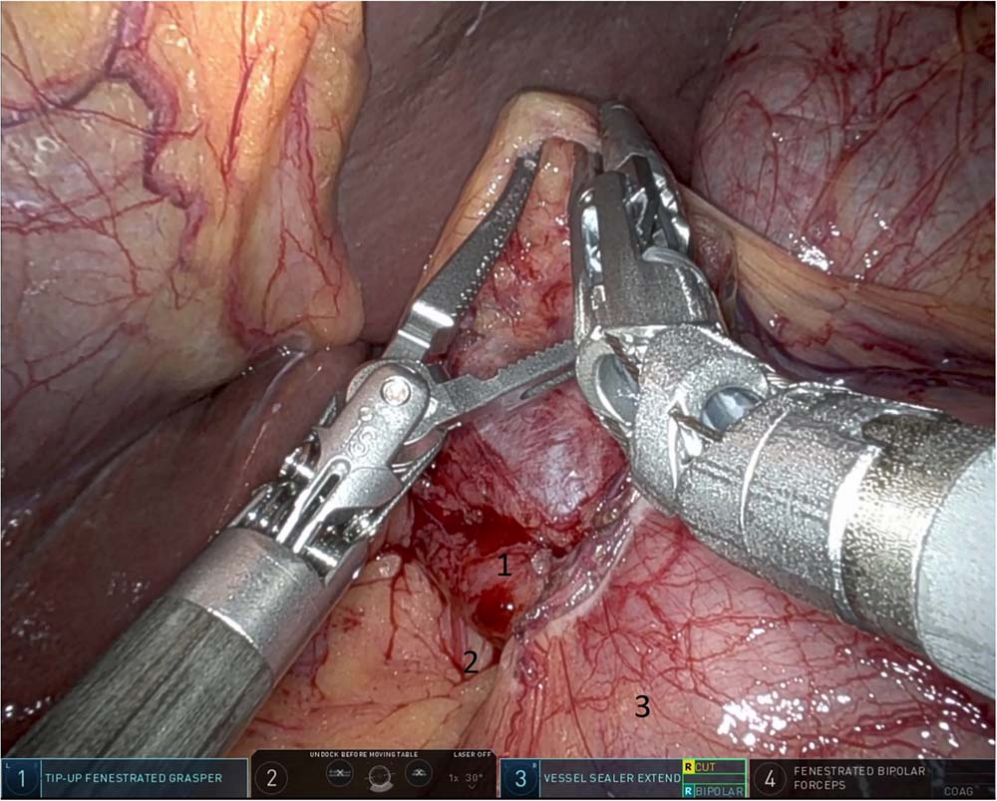
Dissection anterior to the hepatoduodenal ligament (1: portal vein, 2: foramen of Winslow, 3:first part of the duodenum).

**Figure 6:**
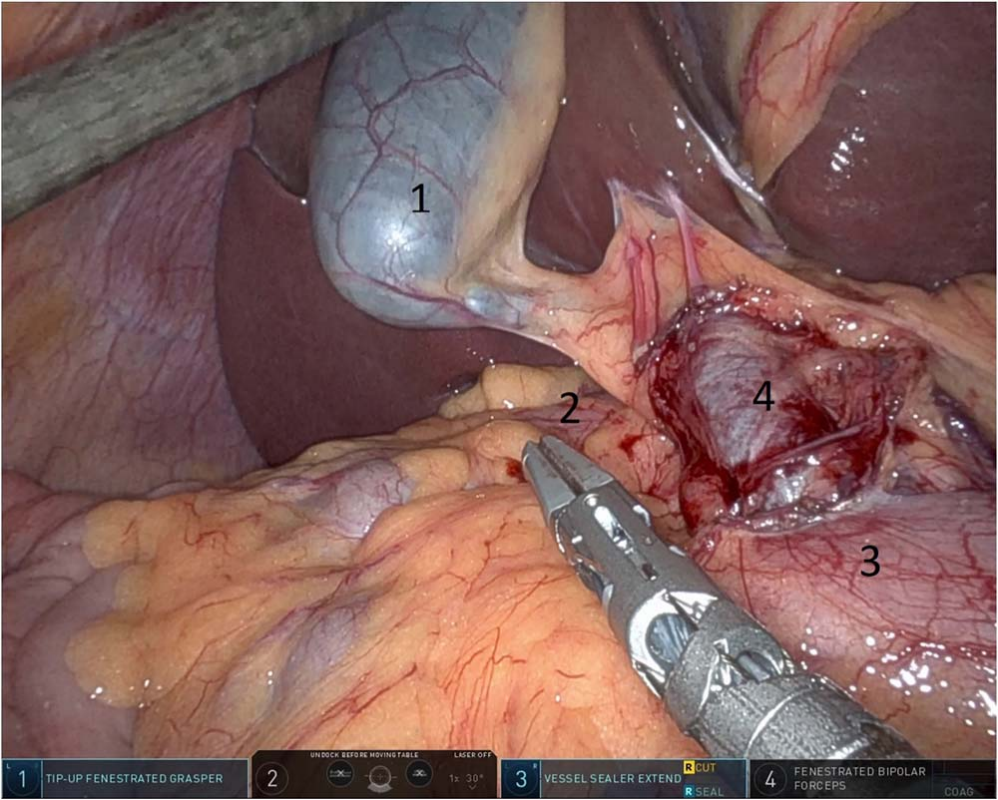
Gallbladder was retracted to the patient's right upper quadrant which allowed for the retraction of the foramen of Winslow and anterior mobilization of it (1: gall bladder, 2: herniating colon, 3: first part of the duodenum, 4: portal vein).

**Figure 7:**
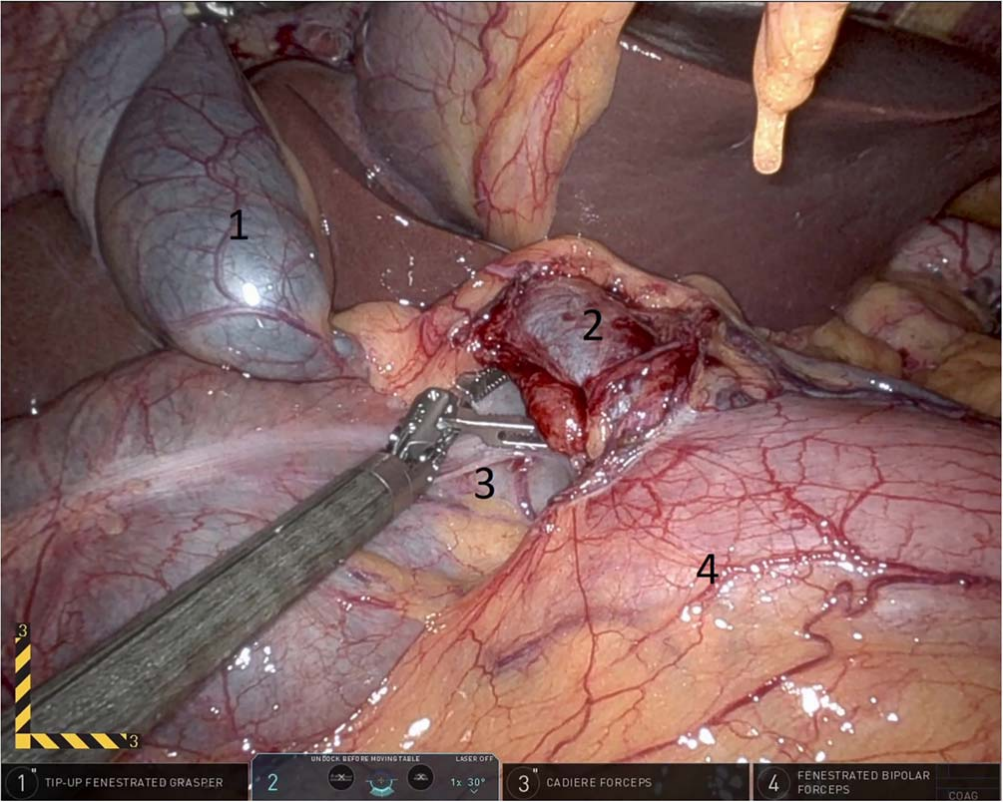
Elevating hepato-duodenal ligament structures to allow reduction of herniating colon (1: gall bladder, 2: portal vein, 3: herniating colon, 4: first part of the duodenum).

Upon entering the abdominal cavity, dilated bowel loops were observed (Fig. 2), and a significant portion of the colon was found herniated through the foramen of Winslow (Fig. 3). The herniated colon was covered by the pars flaccida (Fig. 4) (Video S1). Careful dissection was performed anterior to the hepatoduodenal ligament (Fig. 5). The gall bladder was retracted to the patient's right upper quadrant, which allowed for the retraction of the foramen of Winslow (Fig. 6) (Video S1). After carefully placing the tip of the instrument posterior to the portal vein and gently elevating it, the herniated colon was successfully reduced (Fig. 7) (Video S1).

**Figure 8:**
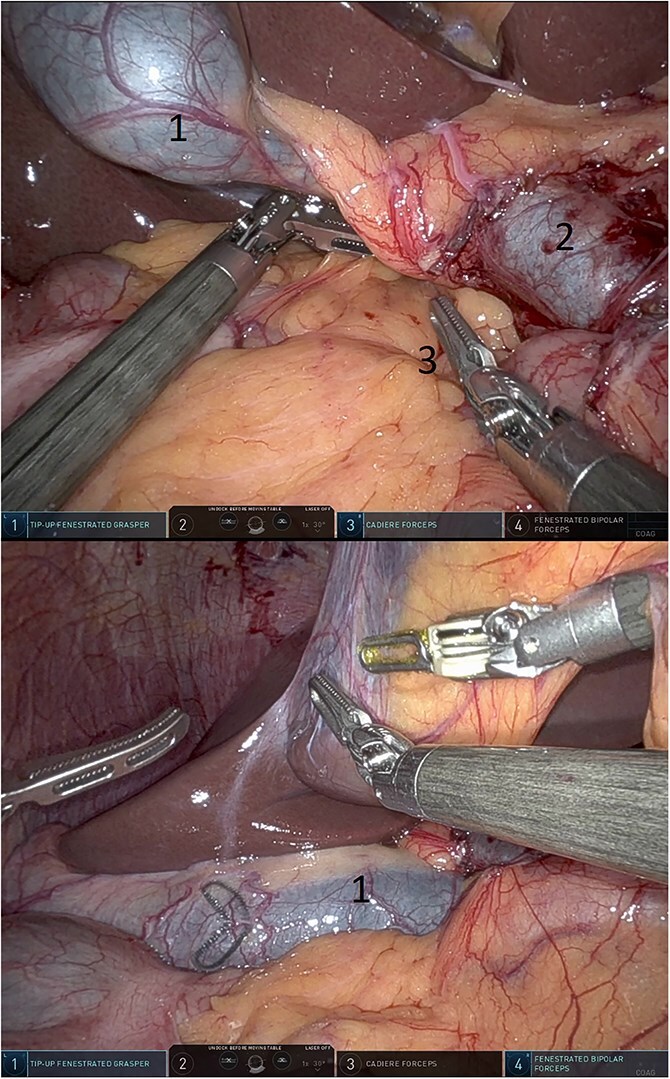
Omentum was placed into the defect (above) and gall bladder fell into the defect (below) (1: gall bladder, 2: portal vein, 3: omentum).

Following reduction, the colon was carefully inspected to assess its viability. Although placement of a suture was considered, the close proximity of the hepatoduodenal structures posed a risk. Therefore, a piece of omentum was placed into the defect to serve as a barrier (Fig. 8). Additionally, after mobilizing the gall bladder, it naturally fell into the foramen of Winslow, potentially acting as a physical barrier to reduce the likelihood of recurrence (Fig. 8).

The patient had an uneventful post-operative period; his abdominal pain resolved, he tolerated liquids well, and was discharged home on postoperative day one.

## Discussion

The typical management of a foramen of Winslow hernia is surgical reduction with careful assessment of the herniated bowel for ischemia or gangrene. This can be achieved using laparotomy or minimally invasive surgery [5].

To the best of our knowledge, and based on a review of the available literature, this represents the first reported case of a foramen of Winslow hernia repair performed using robotic-assisted surgery.

Closure of the foramen of Winslow is controversial; some surgeons advise against closing the foramen due to the risk of damage to the nearby common bile duct or other hepatoduodenal structures, while others support closure with caution [1]. However, there are no reported cases of recurrence after repair in general.

Minimal invasive approaches have increasingly been reported in the literature as safe and feasible, with benefits including reduced postoperative pain, shorter hospital stay, and quicker recovery [1, 5]. In our case, robotic assistance allowed enhanced visualization, precise dissection around the portal triad, and facilitated an atraumatic reduction of the herniated colon.

## Supplementary Material

Video_final_rjaf738
